# Fucoidan Inhibits Radiation-Induced Pneumonitis and Lung Fibrosis by Reducing Inflammatory Cytokine Expression in Lung Tissues

**DOI:** 10.3390/md16100392

**Published:** 2018-10-19

**Authors:** Hsin-Hsien Yu, Edward Chengchuan KO, Chia-Lun Chang, Kevin Sheng-Po Yuan, Alexander T. H. Wu, Yan-Shen Shan, Szu-Yuan Wu

**Affiliations:** 1 Institute of Clinical Medicine, College of Medicine, National Cheng Kung University, Tainan 701, Taiwan; klaus610@gmail.com; 2 Division of General Surgery, Department of Surgery, Wan Fang Hospital, Taipei Medical University, Taipei 116, Taiwan; 3 School of Dentistry, College of Dental Medicine, Kaohsiung Medical University, Kaohsiung 807, Taiwan; ko.edward.kaseizen@gmail.com; 4 Division of Oral and Maxillofacial Surgery, Kaohsiung Medical University Hospital, Kaohsiung 807, Taiwan; 5 Department of FUJISOFT Cartilage and Bone Regeneration, Tissue Engineering, The University of Tokyo, Tokyo 113-0033, Japan; 6 Department of Hemato-Oncology, Wan Fang Hospital, Taipei Medical University, Taipei 116, Taiwan; richardch9@hotmail.com; 7 Department of Internal Medicine, School of Medicine, College of Medicine, Taipei Medical University, Taipei 11031, Taiwan; 8 Department of Otorhinolaryngology, Wan Fang Hospital, Taipei Medical University, Taipei 116, Taiwan; dryuank@gmail.com; 9 Ph.D. Program for Translational Medicine, Taipei Medical University, Taipei 11031, Taiwan; chaw1211@tmu.edu.tw; 10 Department of Surgery, National Cheng Kung University Hospital, College of Medicine, National Cheng Kung University, Tainan 701, Taiwan; 11 Department of Radiation Oncology, Wan Fang Hospital, Taipei Medical University, Taipei 116, Taiwan

**Keywords:** radiation pneumonitis, lung fibrosis, fucoidan, cytokine, macrophage, neutrophil

## Abstract

**Purpose**: Radiotherapy is a crucial treatment approach for many types of cancer. Radiation pneumonitis (RP) is one of the major complications in chest irradiation. Fucoidan is a sulfated polysaccharide found mainly in various species of brown seaweed. Recent studies have demonstrated the anti-inflammatory effects of fucoidan. However, no study has reported a well-established prophylactic agent for RP. Therefore, we investigated the effects of fucoidan on RP and radiotherapy (RT)-induced lung fibrosis. **Materials and Methods**: We compared RP and RT-induced fibrosis in lung tissue specimens obtained from irradiated (10 Gy/shot) C57BL/6 mice with or without fucoidan administration (200 mg/kg/day, oral gavage for 14 days). The expression patterns of cytokines in the pleural fluid were determined using a cytokine array and confirmed through enzyme immunoassays. **Results**: Fucoidan administration attenuated RP and RT-induced fibrosis in lung tissues. Decreased neutrophil and macrophage accumulation was observed in irradiated lung tissues, and radiation-induced lung fibrosis, as demonstrated by Masson trichrome staining, was attenuated. We investigated the expression patterns of inflammatory cytokines in the irradiated lung pleural fluid through the protein array; results revealed that fucoidan administration changed the expression patterns of inflammatory cytokines in irradiated lung tissues. Furthermore, the expression levels of TIMP-1, CXCL1, MCP-1, MIP-2, and interleukin-1Ra were substantially enhanced in the pleural fluid, but fucoidan administration significantly reduced their expression. **Conclusions**: Fucoidan changes the expression patterns of inflammatory cytokines, which may consequently attenuate RP and RT-induced lung fibrosis.

## 1. Introduction

Currently, radiotherapy (RT) is an important approach for treating tumors [1]. The treatment protocol can include curative, adjuvant, neoadjuvant, therapeutic, or palliative therapy, depending on the tumor type, location, and stage as well as the health status of patients [2,3]. Radiation pneumonitis (RP) is the acute manifestation of radiation-induced lung disease and is relatively common following RT for chest wall or intrathoracic malignancies [4]. The acute phase typically occurs between 4 and 12 weeks following the completion of the RT course; however, it may occur as early as 1 week after RT, particularly in patients receiving a high total dose or concurrent chemotherapy [5]. The late phase is characterized by lung fibrosis that occurs as early as 6 months following RT and can progressively continue for several years [6]. Typical symptoms include cough, dyspnea, low-grade fever, chest discomfort, and pleuritic pain. Moreover, the pathology of RP reflects an acute response of the lung to radiation and includes loss of type I pneumocytes and increased capillary permeability that result in interstitial and alveolar edema and the ingress of inflammatory cells into alveolar spaces. Immune-mediated lymphocytic alveolitis has been postulated as the underlying cause of changes in nonirradiated lung tissues [7,8]. Cytokines have been found to play crucial roles in RP and late RT-induced lung fibrosis [9,10,11].

Studies have indicated that preventing or treating RT-induced lung injury is difficult. Until now, no study has reported well-established prophylactic agents for RT-induced lung injury prevention or treatment. Patients who require chest RT have a risk of RP or lung fibrosis, which exerts acute or long-term effects. Therefore, it is necessary to identify an effective prophylactic agent that not only prevents RT-induced lung injury, but also guides the dose escalation of RT to achieve improved local control and overall survival.

A heparin-like molecule, referred to as fucoidan, containing high percentages of L-fucose and sulfated ester groups and small proportions of D-xylose, D-galactose, D-mannose, and glucuronic acid was found in the cell wall matrix of brown seaweed [12]. Various biological activities, such as antioxidant, anti-inflammatory, antiproliferative, anticancer, and proapoptotic activities, have been observed in brown seaweed [13,14,15]. Furthermore, radioprotective effects of fucoidan were observed in mice treated with total body irradiation [16]. The present study investigated the effects of fucoidan on RP and RT-induced lung fibrosis.

## 2. Material and Methods

### 2.1. Fucoidan Reagent

Fucoidan powder extracted from Sargassum hemiphyllum was obtained from Hi-Q Oligo-fucoidan, a commercial product provided by Hi-Q Marine Biotech International Ltd. (New Taipei City, Taiwan). Fucoidan powder or dextran powder (D9260; Sigma-Aldrich, St. Louis, MO, USA) was dissolved in double-distilled H2O and stirred at 25 °C for 30 min. The dissolved solution was filtered using 0.22-μm sterile filters (Millipore, Billerica, MA, USA). Fucoidan used in this study was obtained through the enzyme hydrolysis of original fucoidan. The average molecular weight, fucose content, and sulfate content of fucoidan used in this study were 0.8 KDa (92.1%), 210.9 ± 3.3 mmol/g, and 38.9% ± 0.4% (*w*/*w*), respectively [17].

### 2.2. Animals and Grouping

Eight-week-old male C57BL/6 mice, weighing approximately 25 g, were used as experimental subjects. These mice were divided into four study groups: sham (mice not treated with lung irradiation but were immobilized), RT (mice treated with lung irradiation and administered double-distilled H_2_O; 200 μL/day, oral gavage for 14 days), RT + fucoidan (mice treated with lung irradiation and administered fucoidan; 200 mg/kg/day in 200 μL, oral gavage for 14 days), and fucoidan (mice administered fucoidan; 200 mg/kg/day in 200 μL, oral gavage for 14 days). RT + dextran (mice treated with lung irradiation and administered dextran; 200 mg/kg/day in 200 μL, oral gavage for 14 days), and fucoidan (mice administered fucoidan; 200 mg/kg/day in 200 μL, oral gavage for 14 days). In the fucoidan or dextran-receiving groups, fucoidan or dextran was administered through oral gavage for 3 days before RT; we defined the first day of fucoidan or dextran administration as day 1. RT was administered on day 3, and fucoidan or dextran was administered for 14 days. To obtain the lung tissue for neutrophil and macrophage identification and the pleural fluid, some mice were sacrificed on day 15. To obtain the lung tissue for fibrosis determination, some mice were sacrificed on day 31. Mice were inhalationally anesthetized with 5% isoflurane (2-chloro-2-(difluoromethoxy)-1,1,1-trifluoro-ethane) for 5 min and then placed in a CO_2_ chamber. A fill rate of approximately 10% to 30% of the chamber volume per minute with 100% CO_2_ was used, and mice were unconscious within 2–3 min. We maintained CO_2_ flow for a minimum of 1 min after their respiration ceased. This study protocol was approved by the Institutional Animal Care and Use Committee at Wan Fang Hospital, Taipei Medical University (No. LAC-2015-0167). All animal experiments were performed in accord with relevant guidelines and regulations.

### 2.3. Mouse Lung Irradiation

C57BL/6 mice were immobilized using a customized harness that allowed for the exposure of the trunk along with the whole lung. Fluoroscopy and computed tomography were conducted to determine the position of the lung, design the radiation field, and calculate the dose distribution. The whole lung was irradiated with a rectangular field. The remainder of the body was shielded 5 half-value layers of lead. A linear accelerator (Elekta, 6-MV photon beam, Crawley, UK) was used to perform whole lung irradiation at 10 Gy.

### 2.4. Lung Tissue Dissection

Irradiated or sham C57/B6 mice were sacrificed at the aforementioned time points. Mice were anesthetized with 5% isoflurane, and their pleural fluid was withdrawn 30 s after administering 0.5 mL of normal saline intrapleurally by using a 23-gauge needle. The pleural fluid was then centrifuged at 3000 rpm for 10 min, and the supernatant was collected to perform cytokine analysis, ELISA, or functional assays. To avoid the damage caused by intrapleural injection, lung tissue samples were collected from another group of mice and subsequently embedded in paraffin for sectioning. To determine the expression of collagen I in the lung tissue, the freshly excised whole left lung lobe tissue was homogenized in liquid nitrogen. The total protein content of this tissue was extracted using a cell lysis buffer (Thermo Fisher Scientific, Waltham, MA, USA) and quantified using the Bio-Rad protein assay (Hercules, CA, USA).

### 2.5. Immunohistochemistry

The paraffin-embedded tissue slides were rehydrated in PBS for 15 min, and endogenous peroxidase activity was inhibited by 3% H_2_O_2_ or methanol for 10 min at room temperature. For blocking, 5% nonfat milk or PBS was added for 30 min at room temperature. The slides were incubated with an anti-F4/80 antibody or anti-Ly6G antibody or control IgG at 1:100 dilution for 16 h at 4 °C (F4/80 [C-7] is a mouse monoclonal antibody raised against amino acids 335–634 mapped within an extracellular domain of F4/80 of mouse origin; sc-377009; Santa Cruz Biotechnology; anti-Ly6G antibody is a monoclonal antibody NIMP-R14 is highly specific for murine Ly-6G [NIMP-R14]; (ab2557); Abcam). A peroxidase-conjugated secondary antibody was incubated for 1 h at room temperature and detected by immersing the slides in 0.06% 3,3′-diaminobenzidine tetrahydrochloride, followed by counterstaining with Gill Hematoxylin V.

### 2.6. Masson Trichrome Staining

Masson trichrome staining is widely used to study muscular pathologies, including tissue fibrosis and myofibroblasts, and also to detect and analyze myofibroblasts in lung biopsies [18,19]. Lung sections were stained with Masson Trichrome (Sigma-Aldrich, Saint Louis, MO, USA).

### 2.7. Real-Time Quantitative Reverse Transcription–Polymerase Chain Reaction

The mRNA expressions of Ly6G and F4/80 in the lung tissue were measured using a fluorescein real-time quantitative reverse transcription–polymerase chain reaction (qRT-PCR) detection system (Light Cycler DNA Master SYBR Green I; Roche Molecular Biochemicals, Indianapolis, IN, USA). Primer pairs used were as follows: GAPDH: 5′-GGGAAGGTGAAGGTCGG-3′ and 5′-TGGACTCCACGACGTACTCAG-3′; Ly6G: 5′-TGGACTCTCACAGAAGCAAAG-3′ and 5′-GCAGAGGTCTTCCTTCCAACA-3′; and F4/80: 5′-CTCTGTGGTCCCACCTTCAT-3′ and 5′-GATGGCCAAGGATCTGAA AA-3′. The amplification program consisted of one cycle of initial incubation at 60 °C for 20 min, followed by 40 cycles of denaturation at 95 °C for 10 s, annealing at 55 °C for 10 s, and extension at 72 °C for 10 s. The relative mRNA level was calculated using the 2^−ΔΔCt^ method. The amount of indicated mRNA was normalized to that of GAPDH mRNA and presented in arbitrary units, with 1 U corresponding to the value of the sham group.

### 2.8. Cytokine Array

A mouse cytokine array (Ary006; Proteome Profiler, R&D Systems, Minneapolis, MN, USA) was used to analyze cytokine expression patterns in the pleural fluid. The pleural fluid was first mixed with a biotinylated detection antibody cocktail at room temperature for 1 h, and the array membrane was blocked with the blocking solution provided by the manufacturer. The membrane was then incubated with the samples overnight at 4 °C on a shaker. After washing, horseradish peroxidase-conjugated streptavidin was added to the membrane for 30 min at room temperature, and signal development was achieved by the addition of commercial chemiluminescence detection reagents. A digital imaging system (Bio Pioneer Tech Co., New Taipei City, Taiwan) was used to detect signals, which were further analyzed using the ImageJ program. The number of specimens, N, was 3 per experimental group, and a mean value was calculated.

### 2.9. Determination of TIMP-1, CXCL1, MCP-1, MIP-2, and Interleukin-1Ra

The protein lysate or serum levels of TIMP-1, CXCL1, MCP-1, MIP-2, and interleukin (IL)-1Ra were determined using enzyme immunoassay (EIA) kits (R&D Systems, Minneapolis, MN, USA). The protein levels of pro-collagen I alpha in the lung tissue lysate were determined using a mouse pro-collagen I alpha 1 ELISA kit (ab210579).

### 2.10. Functional Test of the Pleural Fluid for Type I Collagen Expression in Fibroblasts

NIH-3T3 cells were purchased from the American Type Culture Collection (Rockville, MD, USA). Cells are maintained in DMEM (Life Technologies, New York, NY, USA) supplemented with 10% (*vol*/*vol*) fetal bovine serum plus penicillin–streptomycin under sterile tissue culture conditions and cultured in a humidified atmosphere of 5% CO_2_ and 95% air at 37 °C. NIH3T3 cells were cultured in 24-well plates until they reached a density of 1 × 10^5^ cells/well. To prevent contamination of collagen in the pleural fluid, cells were treated with 100 μL of the pleural fluid obtained from different groups for 1 h. Subsequently, cells were washed, fresh medium was added, and the cell culture supernatant was collected after 24 h. The protein levels of type I collagen secreted into the cell culture supernatant were determined through ELISA.

### 2.11. Statistical Analysis

One-way ANOVA with Tukey’s post hoc test was used to compare data among the groups. All statistical analyses were performed using SPSS for Windows, version 18.0 (SPSS Inc., Chicago, IL, USA). A *p* value of <0.05 was considered statistically significant.

## 3. Results

### 3.1. Fucoidan Attenuated RT-Induced Lung Fibrosis in Irradiated Mouse Lung Tissues

**Figure 1:**
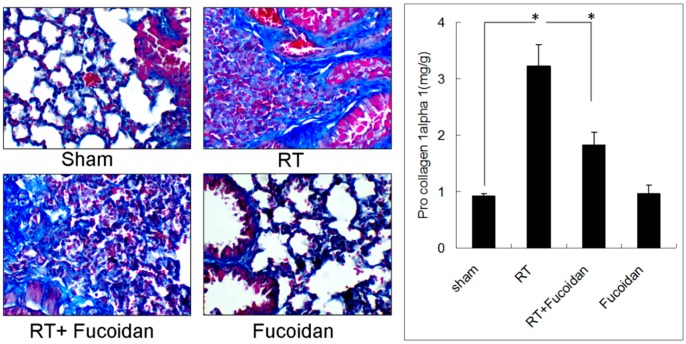
Effects of fucoidan on lung fibrosis in irradiated mouse lung tissues. (**Left** panel): mice from indicated groups were sacrificed on day 31. Representative images of Masson trichrome staining of mouse lung tissues (400×). (**Right** panel): the whole left lobe of the lung of each mouse in each group was homogenized to determine the pro-collagen 1 alpha level through ELISA. N = 5/group. * *p* < 0.05 by one-way ANOVA with Tukey’s post hoc test.

On the basis of the anti-inflammatory effect of fucoidan, we investigated its role in RT-induced lung fibrosis in a mouse model. Mice were divided into four groups, namely sham, RT, RT + fucoidan, and fucoidan, and sacrificed on day 31. We first determined pulmonary fibrosis in each group through Masson trichrome staining (Figure 1, left panel). Type I collagen, which is the major component of pulmonary fibrosis, is encoded by the COL1A1 gene. The COL1A1 gene produces a component of type I collagen, named pro-collagen 1 alpha. Thus, the whole left lobe of the lung was homogenized to determine the pro-collagen 1 alpha level through ELISA (Figure 1, right panel). The results of ELISA revealed increased collagen deposition in the RT group and attenuated collagen deposition in the RT + fucoidan group. The quantitative results of pro-collagen 1 alpha demonstrated that RT significantly enhanced collagen formation in the lung tissue (0.92 ± 0.05 mg/g in the sham group vs. 3.22 ± 0.39 mg/g in the RT group) and fucoidan administration significantly reduced the collagen level in the RT + fucoidan group (1.83 ± 0.23 mg/g). These results indicated that fucoidan attenuated RT-induced lung fibrosis in irradiated mouse lung tissues.

### 3.2. Fucoidan Reduced Neutrophil and Macrophage Infiltration in Irradiated Lung Tissues

**Figure 2:**
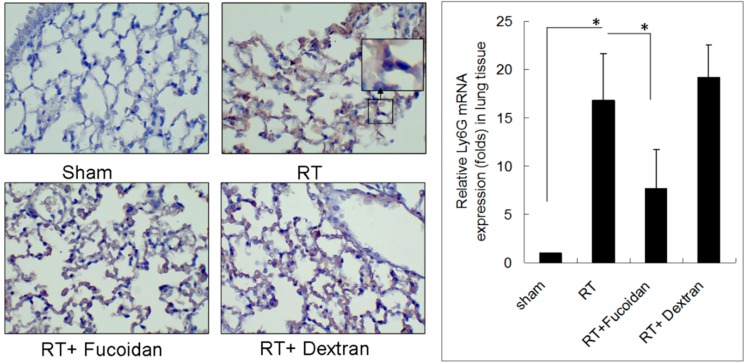
Effects of fucoidan on neutrophil infiltration in irradiated mouse lung tissues. (**Left** panel): mice from indicated groups were sacrificed on day 15. A representative image of neutrophil infiltration in lung tissues (400×). (**Right** panel): the whole left lobe of the lung of each mouse in each group was homogenized for use in qRT-PCR performed for determining the expression level of Ly6G. N = 5/group. * *p* < 0.05 by one-way ANOVA with Tukey’s post hoc test.

**Figure 3:**
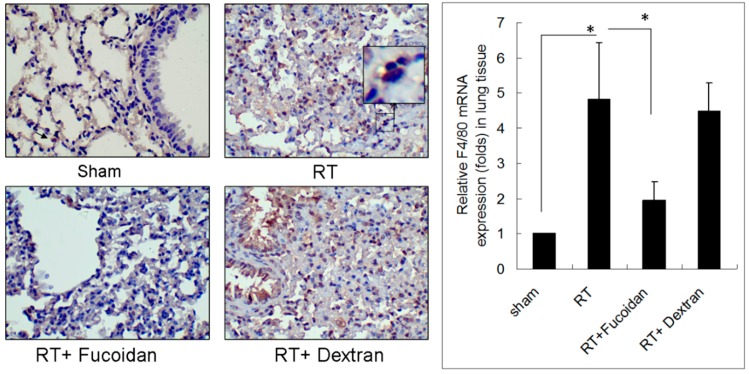
Effects of fucoidan on F4/80 macrophage infiltration in irradiated mouse lung tissues. (**Left** panel): mice from indicated groups were sacrificed on day 15. A representative image of F4/80 macrophage infiltration in lung tissues (400×). (**Right** panel): the whole left lobe of the lung of each mouse in each group was homogenized for use in qRT-PCR performed for determining the expression level of F4/80. N = 5/group. * *p* < 0.05 by one-way ANOVA with Tukey’s post hoc test.

Lung fibrosis is a sequential effect of inflammation. To address the specific effect of Fucoidan, dextran, which is a complex branched glucan is used as polysaccharide control. To observe the phenomenon of neutrophil and macrophage infiltration in lung tissue specimens, mice were divided into four study groups, namely sham, RT, RT + fucoidan, and RT + dextran, and sacrificed on day 15. A part of the lung tissue of each mouse was dissected to prepare paraffin-embedded sections. Neutrophil infiltration was determined through Ly6G antibody staining, which is a specific marker for mouse neutrophils are shown in Figure 2 (left panels). Macrophage infiltration in paraffin-embedded lung tissues was identified through F4/80 antibody staining, which is a specific marker for mouse macrophages (Figure 3, left panels). The whole left lobe of the lung of each mouse in each group was homogenized for use in qRT-PCR performed to determine the expression levels of Ly6G and F4/80, which are specific markers for neutrophils (Figure 2, right panel) and macrophages (Figure 3, right panel), respectively. The quantitative results of Ly6G and F4/80 mRNA demonstrated increased neutrophil (1-fold in the sham group vs. 16.8 ± 4.8-fold in the RT group) and macrophage (1-fold in the sham group vs. 4.8 ± 1.6-fold in the RT group) infiltration in the lung tissues of the RT group. However, the infiltration of neutrophils (7.74 ± 4.01-fold) and macrophages (1.94 ± 0.54-fold) significantly decreased in the RT + fucoidan group but not in RT + dextran group. Meanwhile, Fucoidan administration alone did not change the levels of neutrophil and macrophage infiltration in lung tissues (data not show).

### 3.3. Fucoidan Reduced Cytokine Expression in the Pleural Fluid Obtained from Irradiated Mice

**Figure 4:**
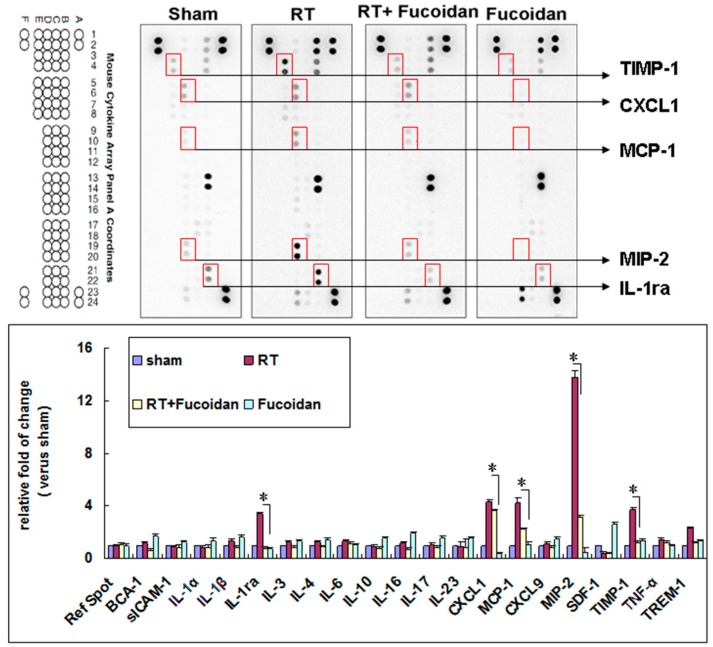
Effects of fucoidan on cytokine expression in the pleural fluid obtained from irradiated mice. (**Upper** panel): pleural fluid was collected from the mice of indicated groups on day 15. Representative image of the cytokine array of pleural fluids. Five spots with obvious changes are indicated. (**Lower** panel): quantitative results of cytokine spots. N = 3/group.

Cytokine expression may reflect the status of inflammation. To examine the expression of cytokines in the lung tissue, mice were divided into four study groups, namely sham, RT, RT + fucoidan, and fucoidan, and sacrificed on day 15. The pleural fluid was collected to analyze the expression patterns of inflammatory cytokines by using a cytokine array. The images of the cytokine array (Figure 4, upper panel) and their corresponding quantitative results demonstrated that fucoidan administration changed the expression patterns of inflammatory cytokines in the pleural fluid obtained from irradiated mice (Figure 4, lower panel). The top five cytokines that were significantly induced in the pleural fluid of the irradiated group than in that of the sham group are as follows: TIMP-1 (3.71 ± 0.11-fold), CXCL1 (4.32 ± 0.12-fold), MCP-1 (4.27 ± 0.13-fold), MIP-2 (13.71 ± 0.14-fold), and IL-1Ra (3.37 ± 0.1-fold). Furthermore, they were significantly reduced in RT + fucoidan group. Additionally, TREM-1 was also induced in the pleural fluid of the irradiated group than in that of the sham group (2.27 ± 0.11-fold) and it was reduced in RT + fucoidan group. On the other hand, by comparing with sham group, the quantitative result revealed that SDF-1/CXCL12 (2.57 ± 0.13-fold) and IL-16 (1.93 ± 0.1-fold) were induced in the pleural fluid of the fucoidan administration only group. However, the expression of SDF-1/CXCL12 (0.37 ± 0.12-fold) and IL-16 (0.68 ± 0.08-fold) in RT + fucoidan group were low.

### 3.4. Fucoidan Reduced TIMP-1, CXCL1, MCP-1, MIP-2, and IL-1Ra Expression in the Pleural Fluid Obtained from Irradiated Mice

**Figure 5:**
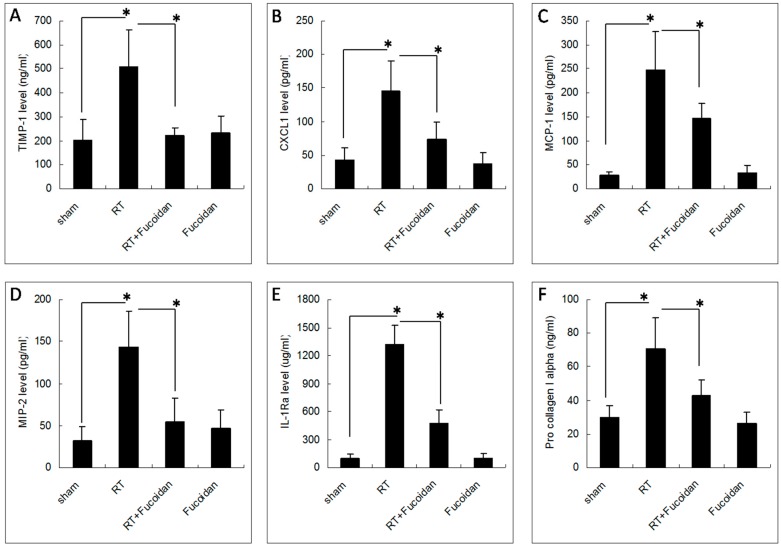
Effects of fucoidan on cytokine levels in the pleural fluid obtained from irradiated mice. The pleural fluid was collected from the mice of indicated groups on day 15. The expression levels of (**A**) TIMP-1, (**B**) CXCL1, (**C**) MCP-1, (**D**) MIP-2, and (**E**) IL-1ra were determined through enzyme immunoassays. Data are compared between indicated groups. * *p* < 0.05. N = 10/group. (**F**) NIH-3T3 cells were treated with the pleural fluid obtained from mice in the sham, RT, RT + fucoidan, and fucoidan groups for 1 h prior to the addition of fresh culture medium and then incubated for another 24 h. The cell culture supernatant was collected for determining the type I collagen level through ELISA. * *p* < 0.05. N = 6/group by one-way ANOVA with Tukey’s post hoc test.

On the basis of the findings of the cytokine array described in Figure 4, we chose the following top five cytokines that were significantly induced in the sham group than in the RT groups: TIMP-1, CXCL1, MCP-1, MIP-2, and IL-1Ra. We determined the concentrations of these cytokines in the pleural fluid of mice in each group through EIA. The results revealed that the expression levels of TIMP-1 (203.1 ± 84.8 ng/mL in the sham group vs. 509.7 ± 151.1 ng/mL in the RT group; Figure 5A), CXCL1 (42.5 ± 17.9 pg/mL in the sham group vs. 145.8 ± 45 pg/mL in the RT group; Figure 5B), MCP-1 (28.6 ± 6.4 pg/mL in the sham group vs. 246.5 ± 80.4 pg/mL in the RT group; Figure 5C), MIP-2 (31.6 ± 16.8 pg/mL in the sham group vs. 143.2 ± 42.6 pg/mL in the RT group; Figure 5D), and IL-1Ra (98.4 ± 45.3 in the sham group vs. 1319.4 ± 203.4 μg/mL in the RT group; Figure 5E) were significantly enhanced in the collected pleural fluid. However, in the RT + fucoidan group, the levels of TIMP-1 (223.1 ± 30.7 ng/mL), CXCL1 (73.3 ± 26.4 pg/mL), MCP-1 (146.4 ± 30.8 pg/mL), MIP-2 (55 ± 27.3 pg/mL), and IL-1Ra (474.4 ± 146.7 μg/mL) were significantly decreased. In order to examine the effect of the cytokines induced by RT in the pleural fluid on fibroblasts and to assess fibrosis induction capability, NIH-3T3 cells, which is a well-known mouse fibroblast cell line, were treated with the pleural fluid obtained from mice in the sham, RT, RT + fucoidan, and fucoidan groups. The pro-collagen 1 alpha level in the cell culture supernatant was determined through ELISA (Figure 5F). The results revealed that, compared with the sham group, the pleural fluid obtained from mice in the RT group significantly increased the type I collagen level in the culture supernatant of NIH-3T3 cells (29.9 ± 6.9 ng/mL in the sham group vs. 70.6 ± 18.4 ng/mL in the RT group). However, compared with the RT group, the type I collagen level was decreased in the cell culture supernatant of NIH-3T3 cells treated with the pleural fluid obtained from mice in the RT + fucoidan group (42.8 ± 9.3 ng/mL).

**Figure 6:**
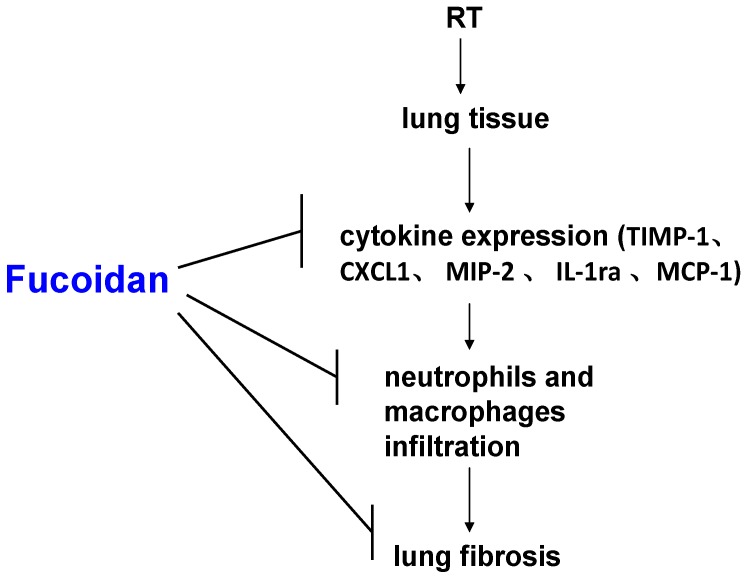
Schematic of possible molecular mechanisms underlying the preventive effects of fucoidan on radiotherapy (RT)-induced lung fibrosis mediated through the reduction of neutrophil and macrophage infiltration and alteration in cytokine expression patterns in lung tissues.

This study demonstrated that fucoidan administration in irradiated mice attenuated cytokine (TIMP-1, CXCL1, MCP-1, MIP-2, and IL-1Ra) expression in the collected pleural fluid and reduced pleural fluid-induced collagen expression in fibroblasts, which were correlated with the reduction of neutrophil and macrophage infiltration in lung tissues; macrophage and neutrophil infiltration may lead to RP and RT-induced lung fibrosis (Figure 6). This result may facilitate the design of fucoidan-based therapeutic approaches for preventing RP in clinical practice.

## 4. Discussion

According to a clinical description, RP occurs in the latent period. However, a molecular biology study revealed a cascade of events occurring immediately after injury and continuing up to the overall occurrence of clinical symptoms such as cough, dyspnea, low-grade fever, chest discomfort, and pleuritic pain [11]. Oxidative stress may cause DNA-damaging injuries; under such conditions, the immediate pulmonary response to radiation-induced injury is similar to many conventional wound healing responses. However, it remains unclear why radiation-induced lung injury is not completely repaired and resolved, with lung tissues consequently entering a progressive and dysregulated process that concludes in the late stage of RP and lung fibrosis. These injuries are now considered to be the result of a complex, structural interaction between multiple cell types, which is initiated and perpetuated through intercell and intracell signaling transduction pathways [20].

A previous study [21] investigated the tissue distribution of 100–200 mg/kg/day of fucoidan after intragastric administration in rats and reported considerable heterogeneity. Fucoidan preferentially accumulated in the kidneys (AUC0-t = 10.74 µg·h/g and Cmax = 1.23 µg/g after 5 h), spleen (AUC0-t = 6.89 µg·h/g and Cmax = 0.78 µg/g after 3 h), and liver (AUC0-t = 3.26 µg·h/g and Cmax = 0.53 µg/g after 2 h) and showed relatively long absorption time and extended circulation in the blood, with a mean residence time of 6.79 h. Although data regarding the fucoidan concentration in the lungs are not available, a study showed the anticancer effect of fucoidan on the lung tissue in vivo [22]. We did not perform a dose-dependent study before. The oral gavage dose administered in the current study was based on the dose suggested in previous studies (200 mg/kg) [23,24,25]. In addition, lung fibrosis is a sequential effect of inflammation. Because fucoidan may attenuate RT-induced inflammation and then reduce lung fibrosis, we examined the effects of fucoidan treatment only for 2 weeks, rather than for 3–4 weeks. We evaluated the phenomena of neutrophil and macrophage infiltration in lung tissue specimens.

RT techniques applied to patients with thorax-related malignancies have resulted in increased lung morbidity and mortality [26,27,28,29,30]. Such therapies involved the exposure of large areas of the heart or lungs to high radiation doses [2,3,4,29]. Although new treatment techniques have reduced both the dose of RT and the area of the heart or lungs exposed to the RT field, it is not clear whether the risk of late complications has been reduced in magnitude or has been merely delayed with regard to the onset time [27,28,29,30]. RT doses to the chest field, such as in breast, esophageal, or lung cancer, might be usually limited by lung or heart tolerance [26,27,28,29]. Furthermore, improved local control or overall survival through RT dose escalation is difficult because of the intolerance of normal tissues [26,27,28,29]. After a long-term follow-up, the grade of acute radiation-induced injury can often escalate to late radiation-induced injury, resulting in long-term RT-related complications [31]. In addition, similar to other organs, recovery from the acute effects of RT in the lungs does not always correlate with the prevention of late critical effects, including fibrosis, which contribute to organ failure [31]. An interventional agent, such as fucoidan, that can protect normal organ tissues from the acute and late effects of RT is crucial. The possibility of dose escalation for improved local control is important [32]. However, RT-induced toxicity in organs, such as the lungs, heart, or gastrointestinal tract, would be problematic in dose escalation because of the intolerance of normal tissues to RT [26,27,28,29]. Until now, no prospective controlled studies have evaluated the efficacy of any preventive drugs or therapies for RP in humans. This study is the first to evaluate the preventive effects of fucoidan on RP and RT-induced lung fibrosis.

In the present study, fucoidan attenuated RT-induced lung fibrosis in irradiated mouse lung tissues on day 31 (Figure 1). Radiation-induced lung injury results from the combination of direct cytotoxicity on normal lung tissues, and more importantly, from the development of fibrosis triggered by radiation-induced cellular signal transduction. The cytotoxic effects are mainly caused by DNA damage that leads to clonogenic death of normal lung epithelial cells, in addition to apoptotic pathways induced by radiation. Fucoidan significantly reduced neutrophil and macrophage infiltration in irradiated mouse lung tissues (Figure 2 and Figure 3). A previous study reported that macrophages may also be present within the alveolar space; these macrophages are migratory cells from the bone marrow and act as a source of numerous cytokines, including IL-6 and TNF-α, to the lungs [33]. In our study, fucoidan attenuated RP and RT-induced lung fibrosis by reducing neutrophil and macrophage infiltration (Figure 6).

Several cytokines are upregulated following lung irradiation and together mediate the corresponding pathological changes. A previous clinical study demonstrated that RT induced the expression of transforming growth factor-beta 1 (TGF-β1), which induced fibroblast collagen deposition [34]. The plasma TGF-β1 level at the end of an RT course was observed to be a predictor of the risk of pneumonitis [34]. However, a subsequent clinical trial revealed no predictable pattern of TGF-β1 changes in individuals with and without radiation-induced lung injury [35]. The proinflammatory cytokines tumor necrosis factor-alpha (TNF-α) and IL-1α have been observed to be upregulated immediately following irradiation. The IL-6 concentration increases following RT, and an elevated pretreatment plasma IL-6 concentration has been shown to be correlated with an increased risk of radiation-induced lung injury [11,36]. However, in this study, we observed no significant differences in IL-1α, TNFα, or IL-6 levels between the sham and RT groups (Figure 4). The discrepancies in results might be due to different sites or the timing of sample collection in different studies. In our study, we collected the pleural fluid from mice of indicated groups on day 15, whereas in previous studies, samples were obtained from human plasma irradiated on day 1 [10,11,35,36]. In our study, the levels of TIMP-1, CXCL1, MCP-1, MIP-2, and IL-1ra were elevated in irradiated mice, which were reduced by fucoidan on day 15. The levels of TIMP-1, CXCL1, MCP-1, MIP-2, and IL-1ra were also determined through EIAs (Figure 5). Fucoidan reduced the expression of TIMP-1, CXCL1, MCP-1, MIP-2, and IL-1ra in the pleural fluid obtained from irradiated mice on day 15. The decreased expression of TIMP-1, CXCL1, MCP-1, MIP-2, and IL-1ra in the pleural fluid obtained from irradiated mice on day 15 was proportional to RT-induced lung fibrosis in mice from the indicated groups that were sacrificed on day 31 (Figure 1). In our study, the increased expression of TIMP-1, CXCL1, MCP-1, MIP-2, and IL-1ra was strongly associated with RT-induced lung fibrosis through the induction of alternatively activated macrophages and neutrophils [37,38,39,40,41,42,43,44,45]. In our current animal model, the pleural fluid was collected on day 15, and mice were sacrificed on day 31. These findings are consistent with late RT-induced lung injuries, such as lung fibrosis (Figure 1). The present results indicated that fucoidan might attenuate RT-induced lung fibrosis by reducing inflammatory cytokine expression and macrophage and neutrophil infiltration. In addition, the mechanism and role of fucoidan on the SDF-1/CXCL12 and IL-16 expression in normal lung tissue need to be further investigated.

Figure 6 presents a brief schematic of the possible molecular mechanism underlying the preventive effects of fucoidan on RT-induced lung fibrosis mediated through the reduction of inflammatory cytokine expression and neutrophil and macrophage infiltration in lung tissues. The strength of our study is that it is the first study to demonstrate the preventive effects of fucoidan on RP and RT-induced lung fibrosis. This finding can be considered in future clinical practice and randomized controlled studies. Therefore, to obtain crucial information on population specificity and disease occurrence, a large-scale randomized trial comparing the effects of fucoidan on carefully selected patients receiving chest irradiation is essential.

## 5. Conclusions

In our study, fucoidan changed the expression patterns of inflammatory cytokines, which may consequently attenuate RP and RT-induced lung fibrosis. Fucoidan can be a potential therapeutic agent for RP attenuation or prevention.

## Abbreviations

RT	Radiotherapy
RP	Radiation Pneumonitis
H&E	Hematoxylin and Eosin
EIAs	Enzyme Immunoassays
DAKO	Diaminobenzidine Tetrahydrochloride
ACEI	Angiotensin-Converting Enzyme Inhibitor
TGF-β1	Transforming Growth Factor-Beta 1
TNF-α	Tumor Necrosis Factor-Alpha
IL-1α	Interleukin 1-Alpha
IL-6	Interleukin 6
